# Non-additive effect of the DNA methylation inhibitor, 5-Aza-dC, and glass as a culture surface on osteogenic differentiation

**DOI:** 10.1016/j.heliyon.2022.e12433

**Published:** 2022-12-17

**Authors:** Latifa Alghfeli, Divyasree Parambath, Loaa A. Tag Eldeen, Ibrahim El-Serafi, Ahmed T. El-Serafi

**Affiliations:** a Research Institute for Medical and Health Sciences, University of Sharjah, United Arab Emirates; b Medical Biochemistry and Molecular Biology Department, Faculty of Medicine, Suez Canal University, Egypt; c Basic Medical Sciences Department, College of Medicine, Ajman University, United Arab Emirates; d Department of Biochemistry, Faculty of Medicine, Port-Said University, Egypt; e Department of Biomedical and Clinical Sciences, Linköping University, Sweden

**Keywords:** Stem cells, Regenerative medicine, Osteogenesis, Bone, miRNA, Glass, Biomaterial, Epigenetic modifier, Epigenetics

## Abstract

The clinical need for bone regenerative solutions is expanding with increasing life expectancy and escalating incidence of accidents. Several strategies are being investigated to enhance the osteogenic differentiation of stem cells. We previously reported two different approaches for this purpose, in monolayer and three-dimensional cell culture. The first approach was based on pretreating cells with 5-Aza-dC, a DNA methylation inhibitor, before the applying the differentiation media. The second approach was based on culturing cells on a glass surface during differentiation. In this study, we investigated the potential effect of combining both methods. Our results suggested that both approaches were associated with decreasing global DNA methylation levels. Cells cultured as a monolayer on glass surface showed enhancement in alkaline phosphatase activity at day 10, while 5-Aza-dC pretreatment enhanced the activity at day 5, irrespective of the culture surface. In three-dimensional pellet culture, 5-Aza-dC pretreatment enhanced osteogenesis through *Runx-2* and *TGF-β1* upregulation while the glass surface induced *Osterix*.

Furthermore, pellets cultured on glass showed upregulation of a group of miRNAs, including pro-osteogenesis miR- 20a and miR -148b and anti-osteogenesis miR -125b, miR -31, miR -138, and miR -133a. Interestingly, 5-Aza-dC was not associated with a change of miRNAs in cells cultured on tissue culture plastic but reverted the upregulated miRNAs on the glass to the basal level. This study confirms the two approaches for enhancing osteogenic differentiation and contradicts their combination.

## 1 Introduction

The increasing global incidence of skeletal disorders highlights the necessity of an effective procedure for bone regenerative solution. The main reasons for skeletal disorders include aging, trauma, and bone and joint diseases. Reduction in bony mass or bone integrity is always associated with declining quality of life. Cell-based tissue engineering is a promising approach for bone regeneration that can replace autologous bone grafting [1]. In this approach, osteogenically induced stem cells are implanted into the defective region of the bone. The cells are expected to undergo further differentiation, secrete osteo-tropic factors, and recruit osteoblast progenitor cells that support and improve bone healing. Therefore, optimizing the osteoblastic differentiation potential of cells is crucial. Mesenchymal stem cells (MSCs) are suitable for cell-based bone engineering due to their known capacity for osteogenic differentiation [2, 3]. However, several studies showed that osteogenic differentiation of MSCs is restricted, to some extent, through repressive epigenetic mechanisms [4].

DNA methylation is an epigenetic modification previously correlated to osteoblastic differentiation, with particular consideration to the expression of key osteogenic markers runt-related transcription factor 2 (Runx2), Osterix (OSX), and alkaline phosphatase (ALP) [5, 6]. The transcription of such genes is negatively correlated with the methylation level within their promoter region [7].

DNA methylation inhibitors, such as 5- azacytidine and 5- aza -2-deoxycytidine, were reported to facilitate the differentiation of MSCs to osteoblasts via upregulating the expression of different osteogenic genes [8]. On the other hand, micro RNAs (miRNAs) are noncoding single-stranded RNAs that exert regulatory effects on gene expression at the post-transcriptional level. miRNAs bind to complementary sequences at the 3′ untranslated region (3′UTR) on messenger RNA (mRNA) and, according to the degree of complementarity, block the transcription or induce the degradation of target mRNA [9]. Different miRNAs can regulate the osteoblastic differentiation of MSCs by targeting the main osteogenic transcription factors and the signaling molecules of osteogenesis [10].

We showed earlier that the DNA demethylating agent 5- aza -2-deoxycytidine (5-Aza-dC) could enhance the differentiation of MSCs into osteoblast-like cells [8]. Recently, we reported the positive effect of glass as a cell culture surface on MSCs differentiation into the osteogenic lineage. The cells showed enhanced proliferation and differentiation and formed a self-assembly three-dimensional (3D) construct rich in the osteogenic matrix [11]. Glass, through inducing histone methylation (H3K4), protects genes from permanent silencing by repelling transcriptional repressors and blocking DNA methylation [12]. In the present study, we investigated the effects of DNA demethylating agent 5-Aza-dC on osteogenic differentiation when the cells were cultured on glass, aiming to provide maximum stimulation for osteogenic differentiation.

## 2 Materials and methods

Chemicals and reagents were purchased from Sigma-Aldrich, St. Louis, USA unless otherwise specified. All the experiments were performed with, at least, three biological replicates.

### 2.1 Cell culture and treatment

MG-63 cell line is commonly used for modeling MSCs, based on their multilineage differentiation ability [11, 13, 14, 15, 16], purchased from ATCC (Manassas, VA, USA). MG-63 cells were cultured in basal media consisting of Dulbecco's Modified Eagle's Medium (DMEM) supplemented with 10% fetal calf serum and penicillin/streptomycin until 50% confluency. To synchronize cell division, cells were serum-starved for 24 h, followed by daily addition of either 1 μM 5-Aza-dC or an equivalent amount of dimethyl sulfoxide (DMSO) as a vehicle or phosphate buffer saline (PBS) as a control for three consecutive days. The cells were recovered in basal media for 48 h. After that, the cells were seeded in a standard 6-well plastic plates or on the top of uncoated coverslips as a glass surface. After 24 h, the media was replaced by osteogenic media, composed of basal media in addition to 10 mM HEPES, 100 μM ascorbate-2-phosphate, and 10 nM dexamethasone that was changed every two days for ten days.

For three-dimensional (3D) pellet culture, 5 × 10^5^ cells were transferred into borosilicate glass or polystyrene (plastic) tubes in 1 ml of osteogenic media and centrifuged for 10 min at 400 *g*. The detailed protocol and photographs of the tubes and pellets can be retrieved from our previous publication [11]. The media was changed every two days for 21 days without disturbing the cell pellet.

### 2.2 Global DNA methylation assessment

Genomic DNA (gDNA) was extracted from the 3D pellets described in our previous study, which shared the same negative control groups [11]. 50 ng of gDNA was used to evaluate the level of methylated cytosine (5-mC) using the Global DNA Methylation Assay Kit (Abcam) according to the manufacturer's instructions. The color developed by this enzyme-linked immunosorbent assay was read at 450 nm and corresponded to the content of 5-methylcytosine. Polynucleotides consisting of 50% cytosine, either methylated or non-methylated, were used as positive and negative controls, respectively.

### 2.3 Molecular characterization of mRNAs and miRNAs expression

Total RNAs (including miRNAs) and gDNA were extracted using All-in-One Purification Kit (Norgen Biotek). At the end of the differentiation phase, the 3D pellets were disintegrated in a 0.6-gauge needle with the lysis buffer, and the lysate was added to a spin column. After centrifugation, mRNA and DNA bound to the column while the flow-through contained miRNAs and proteins. miRNAs were purified using the provided enrichment spin column. mRNAs, gDNA, and miRNAs were eluted following the manufacturer's instructions. mRNA was reverse transcribed into cDNA using the TruScript™ kit (Norgen Biotek). cDNA was quantified spectrophotometrically at 260 nm using Thermo Fisher Scientific NanoDrop 2000. 100 ng cDNA/reaction were added to the SYBR Green reaction mixture (GoTaq® qPCR Master Mix, Promega). Quantitative real-time polymerase chain reaction (q RT-PCR) was carried out using Rotor-Gene Q (Qiagen), and the primer sequences were listed in Table 1. The amplification conditions were initial incubation at 95 °C for 2 min followed by 40 cycles of denaturation at 95 °C for 15 s, annealing at 60 °C for 30 s and extension at 60 °C for 30 s. Target gene expression was normalized to the housekeeping gene, Glyceraldehyde 3-phosphate dehydrogenase (*GAPDH*).

**Table 1:** List of RNA primers sequences used for PCR study.

Genes	Primers Sequence (5′- 3′)
alkaline phosphatase **(*ALP*)** [17]	F: GCCTACCAGCTCATGCATAAC
	R: GAAGTGGGAGTGCTTGTATCT
collagen type 1 (***COL1A1*)** [17]	F: ACTGGTGAGACCTGCGTGTA
	R: CCAGTCTGCTGGTCCATGTA
Osterix (***OSX)*** [18]	F: GGCACAAAGAAGCCGTACTC
	R: TGGGAAAAGGGAGGGTAATC
runt-related transcription factor 2 (***Runx2*)** [18]	F: TCTTCACAAATCCTCCCC
	R: TGGATTAAAAGGACTTGGTG
vascular endothelial growth factor **(*VEGF*)** [19]	F: CCGCAGACGTGTAAATGTTCCT
	R: CGGCTTGTCACATCTGCAAGTA
transforming growth factor beta 1 (***TGF-β1*)** [20]	F: CCCAGCATCTGCAAAGCTC
	R: GTCAATGTACAGCTGCCGCA
glyceraldehyde phosphate dehydrogenase **(*GAPDH*)** [21]	F: CCAGGTGGTCTCCTCTGACTTC
	R: TCATACCAGGAAATGAGCTTGACA

For miRNAs characterization, miRNAs were reverse transcribed into cDNA using miScript II RT Kit (Qiagen). 100 ng cDNA were added to SYBR Green-based PCR master mix from the miScript SYBR Green PCR Kit (Qiagen) with miRNA-specific primers (Table 2). PCR cycles were as follows: initial activation at 95 °C for 15 min, followed by 40 cycles of denaturation at 94 °C for 15 s, annealing at 55 °C for 30 s, and extension at 70 °C for 30 s miRNA expression was normalized against U6 small nuclear RNA. In all experiments, the change in gene expression was calculated according to the ΔΔct method, using the negative control (PBS-treated) group for normalization, as mentioned previously [22].

**Table 2:** List of miRNA primer sequences used for PCR study.

Micro RNA	Primers Sequence (5′- 3′)
**U6** [23]	F: ATTGGAACGATACAGAGAAGA TT
	R: GGA ACG CTT CAC GAA TTT G
**miR-148b** [24]	F: TCAGTGCATCACAGAACTTTGTAA
	R: GCTGTCAACGATACGCTACGT
**miR-138** [25]	F: GCCGCAGCTGGTGTTGTGAAT
	R: GCGAGCACAGAATTAATACGAC
**miR-20a** [26]	F: GCCCGCTAAAGTGCTTATAGTG
	Universal R: GTGCAGGGTCCGAGG
**miR-31** [27]	F: GCCGCAGGCAAGATGCTGGC
	R: CAGTGCAGGGTCC GAGGT
**pre-mir-15b** [28]	F: GGCCTTAAAGTACTGTAGCAGC
	R: CCTTAAATTTCTAGAGCAGC
**miR-125b** [29]	F: CCAGATACTGCGTATGTGTG
	R: GTCACCTGATCCCATCTAAC
**miR-214** [30]	F: AGCCGACAGCAGGCACAGACA
	R: GCTTCGGCAGCACATATACTAAAAT
**miR-133a** [31]	F: TTTGGTCCCCTTCAACCAGCTG
	Universal R: GTGCAGGGTCCGAGG

### 2.4 Alkaline phosphatase (ALP) activity assay

On the 5th and 10th days of differentiation, monolayer cells were rinsed in PBS and fixed with 95% ethanol for 15 min at -20 °C followed by PBS wash. ALP substrate was prepared by adding 400 μl of Naphothol AS-MX phosphate solution, 2.4 mg fast violet B salts, 9.6 ml of distilled water (dH_2_O), and 300 μl were added to each well and incubated for 30 min at 37 °C. As previously described, the reaction was stopped by rinsing the wells with dH_2_O [11]. An average of seven images were analyzed for color intensity by Image J software (NIH, Bethesda, USA).

### 2.5 Cell transfection with miR-214 inhibitor and assessment of osteogenic differentiation

MG-63 cells were transfected with 50 pmol of either miR-214 inhibitor oligonucleotide; 5′-ACUGCCUGUCUGUGCCUGCUGU-3′, or scrambled miRNA oligonucleotide; 5′-CAGUACUUUUGUGUAGUACAA-3’ [32] using Lipofectamine™ 3000 Transfection Reagent (Invitrogen) according to the manufacturer's protocol. Non-transfected cells were considered as the negative control. After 24h, the cell culture media was replaced with osteogenic or basal media and changed every two days. On the 10th day, ALP activity was assessed, as mentioned previously [11].

### 2.6 Statistical analysis

Statistical significance between groups was determined using Two-way ANOVA test. Two-way ANOVA was calculated using GraphPad Prism 9 for macOS, GraphPad Software, San Diego, California USA, followed by Tukey's Honest Significant Difference as a post hoc test. The accepted p-value for significance was less than 0.05. Error bars in all figures represent the standard deviation of the mean.

## 3 Results

### 3.1 The effect of 5-Aza-dC and culture surface on global DNA methylation status

**Figure 1:**
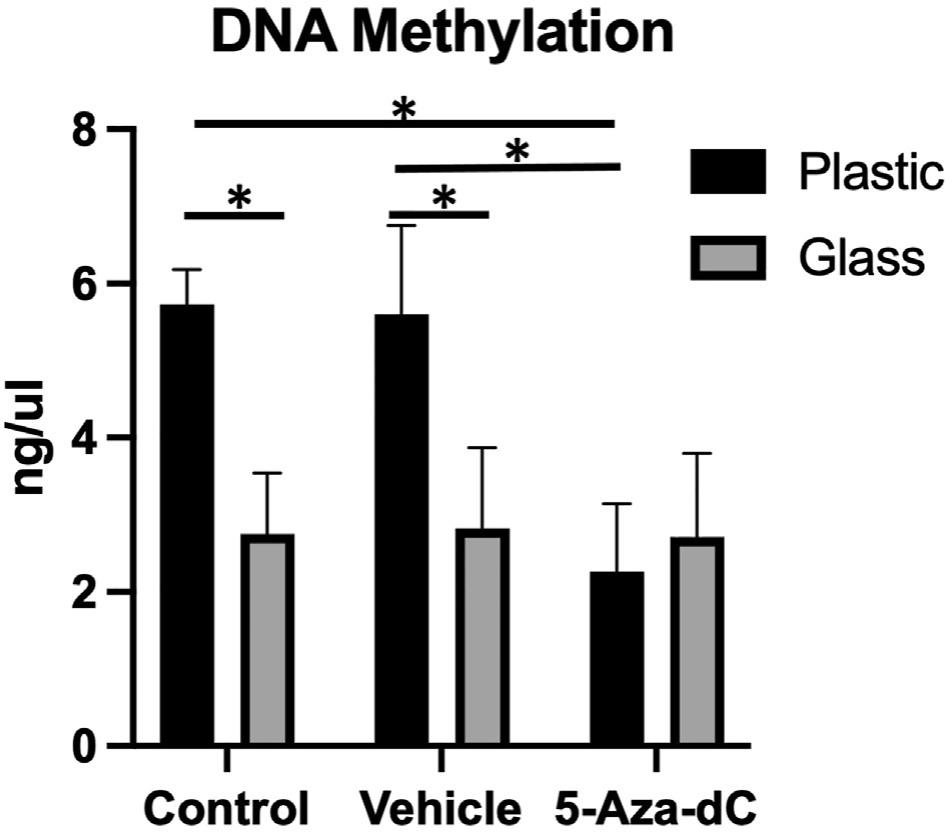
Global DNA methylation level. Quantification of 5-mC content of DNA samples from PBS, DMSO and 5-Aza-dC pretreated cells. Glass decreased the methylation level significantly to almost 50% in the negative control (PBS) and vehicle control (DMSO). Similarly, 5-Aza-dC decreased the DNA methylation level on plastic but had no additive effect on glass. ∗P < 0.05.

The level of 5-methyl cytosine in cells pretreated with 5-Aza-dC and cultured in a plastic tube (2.3 + 0.9 ng/ul) was significantly reduced compared to those pretreated with DMSO (5.6 + 1.2 ng/ul) or PBS (5.7 + 0.5 ng/ul). The pattern of methylation level was different in cells cultured on glass, as all groups showed a similar level of 5-methyl cytosine (PBS, 2.7 + 0.9 ng/ul; DMSO, 2.8 + 1.1 ng/ul; 5-Aza-dC, 2.7 + 1.2 ng/ul). Interestingly, cells cultured on glass and pretreated with PBS or DMSO showed low levels of 5-methyl cytosine compared to those cultured on plastic and comparable to cells cultured on plastic with 5-Aza-dC pretreatment. Furthermore, pretreatment with 5-Aza-dC seems to have no additional effect in the cells cultured on the glass as no cumulative impact was found, and the methylation level was similar to 5-Aza-dC pretreated cells, irrespective of the culture surface (Figure 1). Analysis of variance confirmed the presence of a significant difference between the groups.

### 3.2 The effect of 5-Aza-dC and culture surface on ALP activity

**Figure 2:**
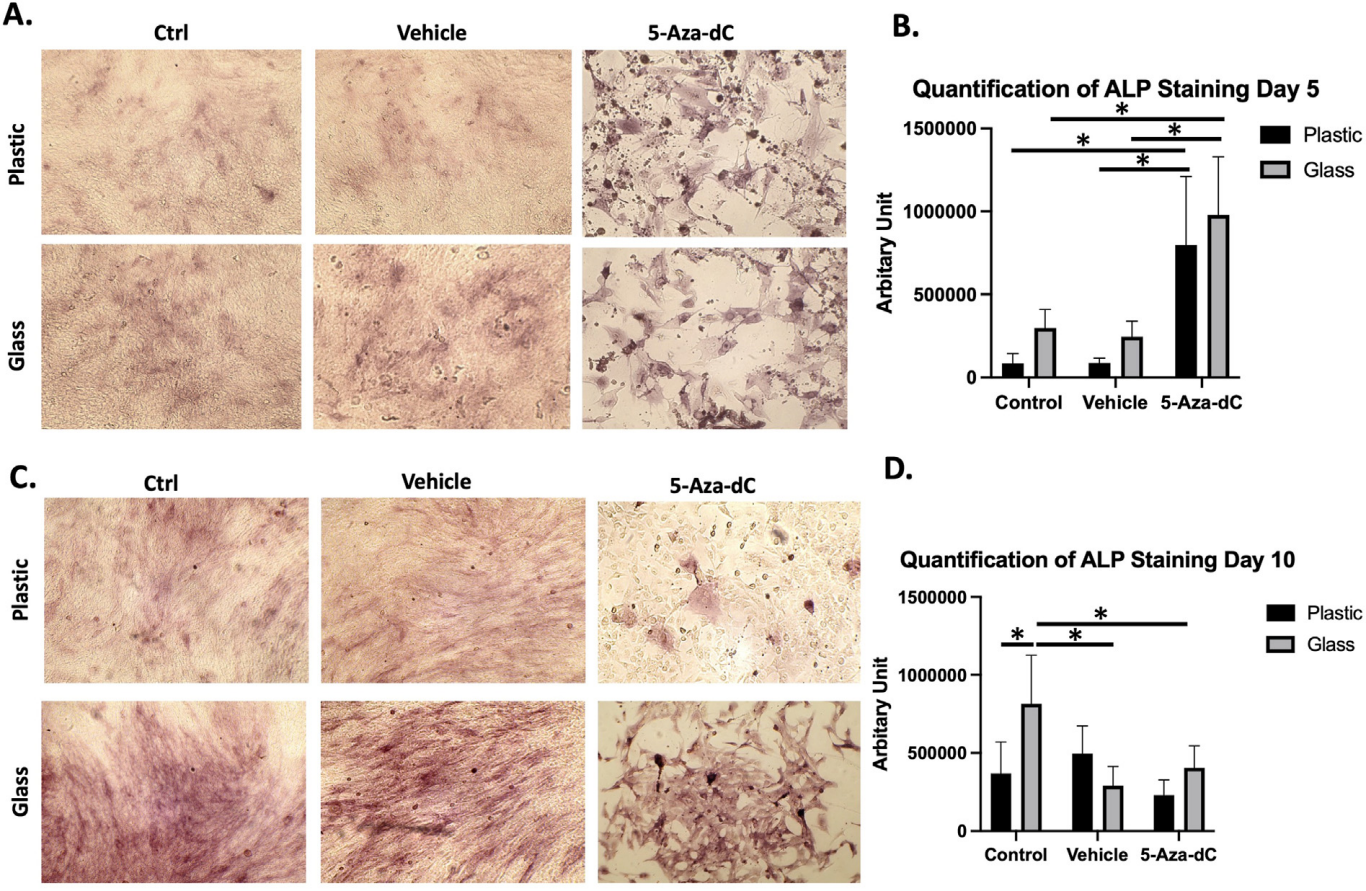
ALP activity on plastic and glass surface. (A) ALP activity (indicated by red stain) at day 5 (B). Quantification of ALP staining at day 5. (C) ALP activity at day 10. (D) Quantification of ALP staining at day 10.

On day 5, monolayer cells cultured on a glass surface showed a trend of higher ALP staining intensity (Figure 2A). However, ALP staining pattern in cells pretreated with 5-Aza-dC was higher than control groups, i.e., cells pretreated with either PBS or DMSO. No significant difference was found between cells pretreated with 5-Aza-dC cultured on the two studied surfaces. However, two-way ANOVA showed significant effect of both materials and treatment among the groups (Figure 2B). On day 10, the intensity of ALP staining was noticeably increased in control samples, whereas ALP was significantly higher in cells cultured on glass (Figure 2C). Interestingly, the intensity of ALP staining was lower in cells treated with 5-Aza-dC compared to day 5, but the intensity was higher in glass compared to plastic. Similar to day 5, two-way ANOVA showed significant effect of both material and treatment among the groups on day 10 (Figure 2D). Overall, ALP activity increased with time in control groups but decreased in the 5-Aza-dC treated group.

### 3.3 The effect of 5-Aza-dC and culture surface on the expression of bone related genes

**Figure 3:**
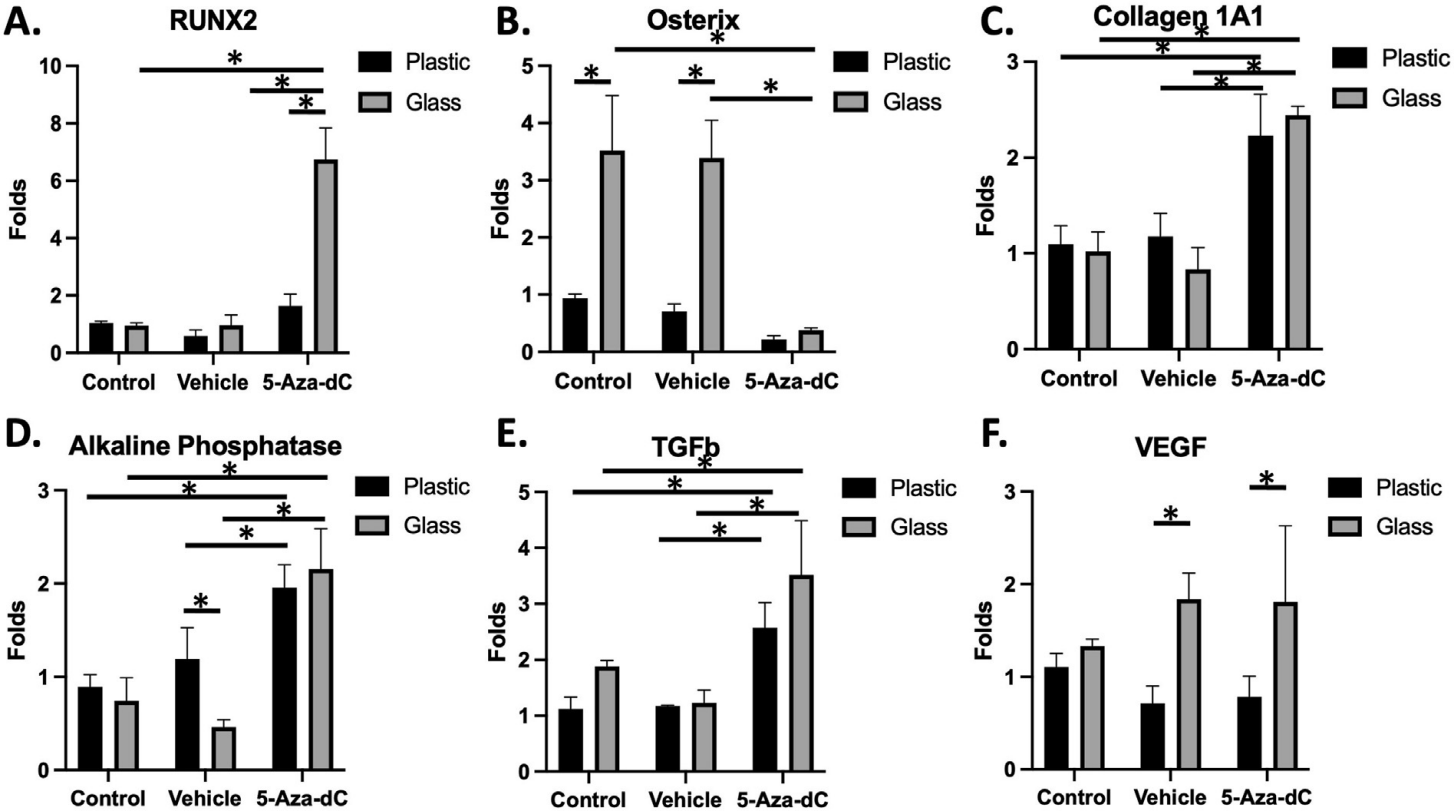
The gene expression pattern for 3D cultured cells in plastic and glass tubes. (A) 5-Aza-dC enhanced the expression of *Runx-2* in glass surface while the glass alone showed no change of expression. (B) The glass surface increased the expression of *OSX* while pretreatment with 5-Aza- dC was associated with its inhibition. (C, D, E) enhancement of the expression of *COL1A1, ALP* and *TGF β1* with 5-Aza-dC pretreatment on both surfaces. (F) Glass increased the expression of *VEGF* in the vehicle and 5-Aza-dC pretreatment group. ∗p < 0.05.

5-Aza-dC pretreatment enhanced the expression of *Runx-2* in cells cultured as 3D pellets in glass tubes (Figure 3A). On contrary, 5-Aza-dC inhibited the expression of *OSX* in cells cultured in both glass and plastic tubes, while the cells cultured on glass surfaces demonstrated higher expression of *OSX* in the control and vehicle groups compared to their plastic counterparts (Figure 3B). 5-Aza-dC enhanced the expression of other osteogenic markers, including *COL1A1* (Figure 3C), *ALP* (Figure 3D), and *TGF β1* (Figure 3E) in comparison to the vehicle (DMSO) and negative control (PBS), irrespective of the surface. However, being on a glass surface increased the expression of *VEGF* compared to plastic in the vehicle as well as in the 5-Aza-dC group (Figure 3F). Two-way analysis of variance confirmed the differences between the groups for all studied genes, as well as the effect of 5-Aza-dC and glass surface on the expression of *Runx-2 and OSX*, the effect of 5-Aza-dC on *ALP, Col1A1* and *TGF β1,* and the effect of glass on *VEGF* expression.

### 3.4 The effect of 5-Aza-dC and culture surface on miRNAs expression

**Figure 4:**
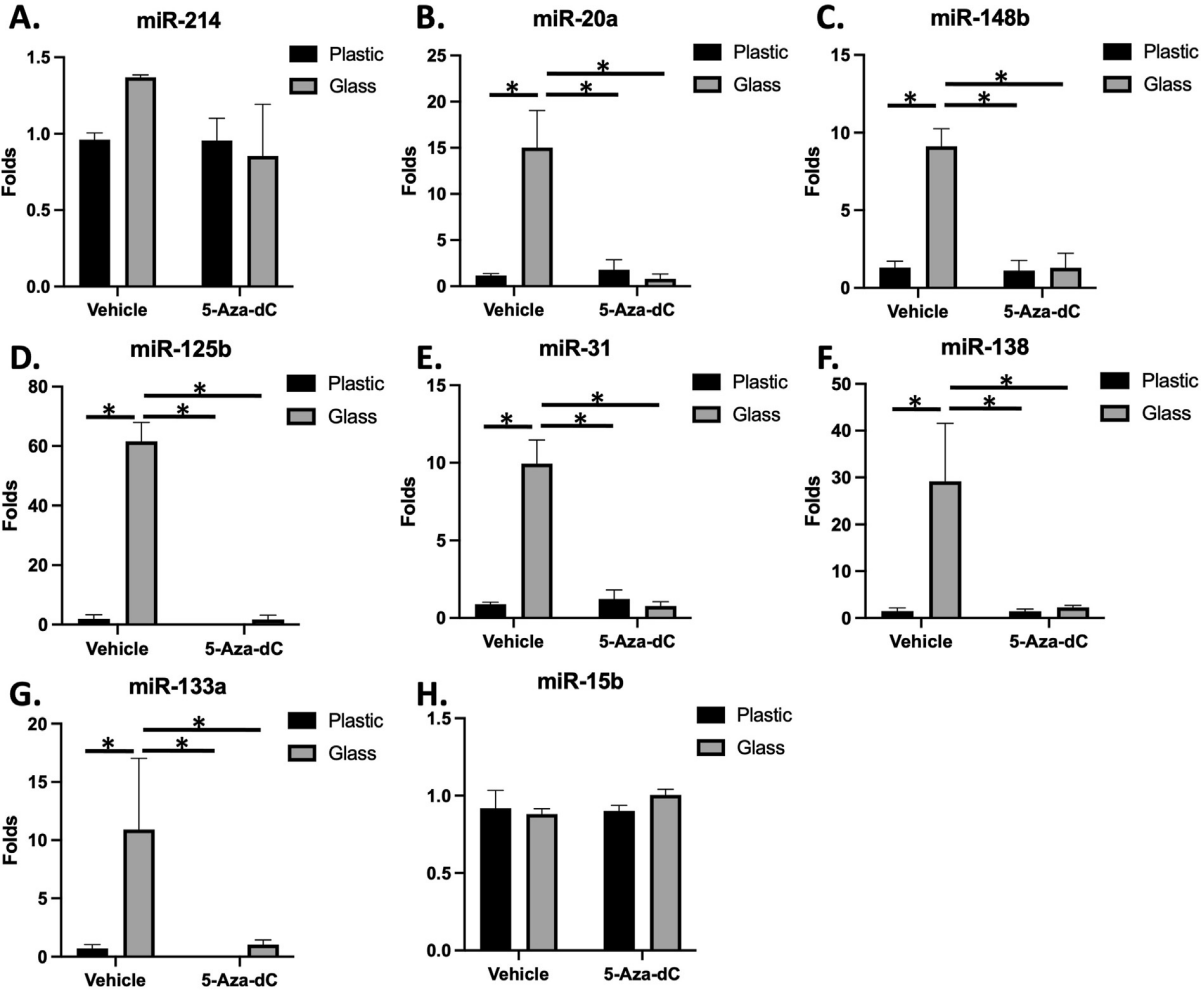
miRNAs expression pattern for 3D cultured cells in plastic and glass tubes. Glass enhanced the expression of miR-20a (B), miR-148b (C), miR-125b (D), miR-31 (E), miR-138 (F) and miR-133a (G). Such an effect was reverted by 5-Aza-dC pretreatment. miR-214 (A) and miR-15b (H) were not affected by the culture surface or 5-Aza-dC pretreatment. ∗p < 0.05.

Most of the studied miRNAs were upregulated in cells cultured as 3D pellets in glass tubes, compared to those cultured in plastic and pretreated with the vehicle (DMSO). This group included miR-20a, miR-148b, miR-125b, miR-31, miR-138, and miR-133a (Figure 4B, C, D, E, F, G).

For cells cultured in glass tubes, 5-Aza-dC decreased the expression of the same group of miRNAs, in comparison to the vehicle. Interestingly, 5-Aza-dC had no effect on the studied miRNAs for cells cultured in plastic tubes.

It is also worth to be noted that the expression of miR-214 and miR-15b (Figure 4A and H) was not affected by the pretreatment or the culture surface.

### 3.5 The effect of miR-214 inhibition on ALP enzyme activity

**Figure 5:**
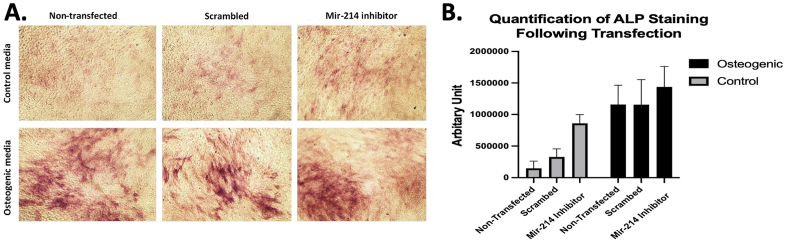
Transfection with miR-214 inhibitor. (A) Cells transfected with miR-214 inhibitor demonstrated higher levels of ALP enzyme activity than non-transfected cells when cultured in control (basal) or osteogenic media. (B) Quantification of ALP staining.

Cells transfected with miR-214 inhibitor showed higher levels of ALP enzyme activity than non-transfected cells or those transfected with scrambled miRNA oligonucleotide. The cells were cultured in osteogenic or basal media. However, ALP activity was more evident in osteogenic than in control media (Figure 5A). Two-way analysis of variants confirmed the statistical significance among the study groups while Tukey's multiple comparisons analysis failed to show statistical significance between the groups in each media type (Figure 5B).

## 4 Discussion

Differentiation of MSCs into mature osteoblasts involves four phases: commitment of MSCs to osteoprogenitors, development of these cells into pre-osteoblasts, expansion of the pre-osteoblasts, and finally, maturation to osteoblasts [33]. The expression of osteoblast-specific markers characterizes each phase. The cells in the first phase express the transcription factor Runx2, the extracellular enzyme ALP, and the extracellular matrix protein, collagen type I [34, 35]. Runx2, a core transcription factor of osteogenesis, binds to osteoblast-specific cis-acting element (OSE2) in osteogenic-related gene promoters, including collagen-1 and ALP [36]. At the next stage, OSX expression increases, while ALP expression declines gradually as the cells mature [37]. The final stages are characterized by high expression of osteocalcin and osteopontin, followed by calcium and phosphate deposition in the extracellular matrix [38, 39]. The phase transition is controlled by synchronization between extracellular ligands related to TGF, BMP and WNT signaling cascades and epigenetic events that direct the expression of key transcription factors [40].

Despite our understanding of these molecular and cytological events, enhancing the osteo-inductive ability of stem cells has been an unmet challenge. To advance bone engineering for tissue regenerative purposes, several approaches were investigated. Epigenetic modifiers and biomaterials are among the most studied modulators of osteogenic differentiation of MSCs. We have shown previously that 5-Aza-dC can enhance the osteogenic differentiation of MSCs when added as a pre-differentiation treatment step [41]. 5-Aza-dC is a DNA methylation inhibitor, which has been shown in this study to decrease the global levels of 5 methyl cytidine. Hypomethylation at genes promoter allows DNA to adopt a conformation that destabilizes nucleosomes, facilitates the binding of transcription factors to gene promotors, and initiates gene transcription [6]. Although the epigenetic modifiers were considered as non-specific agents, accumulating evidence suggests fostered effects on specific genes [4, 42], mainly when applied in a pre-differentiation step for MSCs [15, 16, 41, 43]. In adipogenic differentiation, 5-Aza-dC pretreatment was investigated with MG63. Thirty-three transcription factors and sixteen signaling pathways were upregulated, including MAP kinase and PI3-Akt pathways [16]. Both pathways are involved in the osteogenic differentiation of MSCs [44, 45].

**Figure 6:**
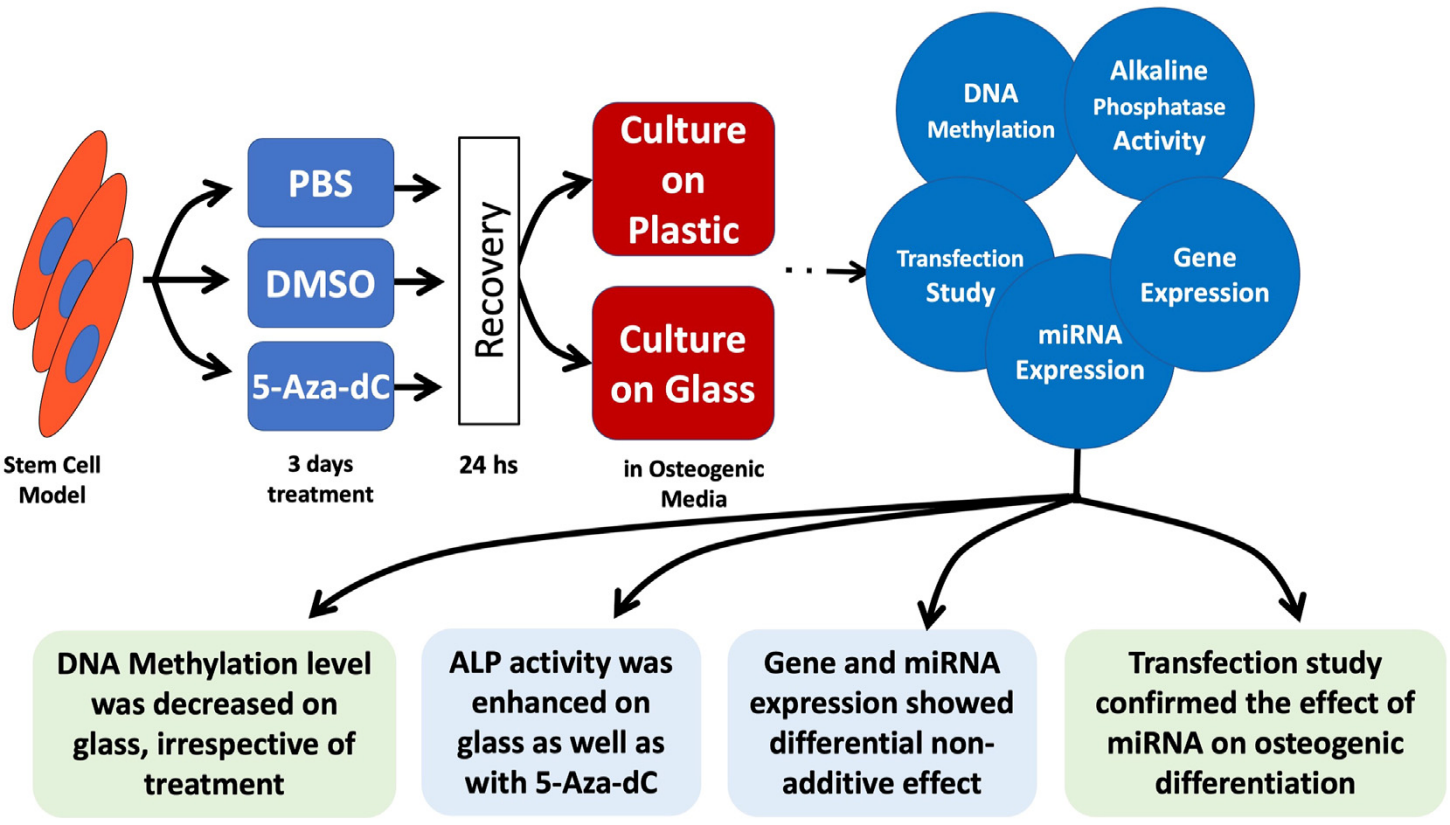
Summary of the study design and main findings.

Recently, we reported that glass as a culture surface drives MSCs into forming 3D osteogenic pellets through a self-assembly approach. These osteogenic constructs showed enhanced molecular and cellular osteogenic biomarkers compared to their counterparts cultured on the plastic surface [11]. Glass has been used as an osteo-inductive material for bone regeneration in orthopedic and dentistry applications for several years [46, 47]. In addition, glass nanoparticles were included as a component of bio-inks to enhance bone tissue engineering [48, 49]. In the present study, we combine the two factors, i.e., epigenetic modifier pretreatment and the culture surface, aiming to obtain the maximum differentiation efficiency. The summary of the study is illustrated in Figure 6.

In agreement with our previous studies, pretreating MSCs with 5-Aza-dC or culturing MSCs on glass boosted their differentiation status, as indicated by enhanced ALP activity and molecular characteristics. However, the studied markers showed no synergistic effect of the two factors for improving differentiation, i.e., epigenetic modifier pretreatment and cell culture surface. Pretreatment with 5-Aza-dC on classical tissue culture plastic was associated with upregulation of the transcription factors *Runx2* and *TGF-Β1*, as well as the downstream markers *ALP* and *COL1AI.* Assis et al. (2021) showed upregulation of Runx2 in periodontal ligament cells similarly to pretreatment with the DNA methyltransferase inhibitor, RG108, as well as the formation of mineralized nodules after 21 days in culture [50]. TGF-β1 signaling affects bone formation by inducing *Runx2* expression through Smad2/3 signal cascade and promoting osteogenic matrix proteins [51]. Thus, *Runx2* upregulation could be explained by the direct effect of 5-Aza-dC or through upregulation of TGF- β or their combination. The upregulation ultimately enhanced the expression of *ALP* and *COL1A1*. This hypothetical explanation needs further testing by blocking various intermediates and investigating the osteogenic differentiation efficiency.

Culturing the cells as 3D pellets in glass tubes was associated with the upregulation of the transcription factors *OSX* and *VEGF*, which have essential roles in promoting osteogenesis. *OSX* upregulation, not *Runx2*, was consistent with our previous characterization of the self-assembly 3D bone constructs cultured on glass [11]. Although OSX is a downstream target of Runx2, it can also be induced through the BMP2 signaling cascade [52]. Alternatively, enhanced *OSX* expression with glass could be related to the global DNA hypomethylation, as the latter can be associated with trimethylation on the 4th lysine of histone 3 (H3K4me3) [53,54]. H3K4me3 forms a loose chromatin structure, inducing the transcription of OSX that increases the expression of the downstream osteoblast markers, such as vitamin D receptor, ALP, COL1A1, and other bone matrix proteins [55, 56]. Interestingly, 5-Aza-dC has been shown to retain gene activity for promotors related to H3K4me3 [57,58].

Similarly, the presence of H3K4me3 adjacent to the *VEGF* promotor region can enhance its expression [59]. Osteoblasts can synthesize VEGF in order to stimulate migration and proliferation of endothelial cells, as effective bone formation requires interaction between angiogenic and osteogenic processes [60, 61]. This data suggests the potential role of glass in enhancing the formation of osteogenic constructs with vascular elements, mimicking the physiological development, and providing a possible advantage in integrating with surrounding tissue upon implantation *in vivo*. Interestingly, 5-Aza-dC downregulated the expression of *OSX*, which could be explained by the inhibition of the Wnt/beta-catenin signaling pathway induced by the DNA demethylation-dependent upregulation of Wnt antagonist [62, 63]. Nevertheless, studying the temporal expression of Runx2 and OSX can help further characterize the differentiation enhancement mechanism for both culturing systems.

miRNAs can be produced in response to the metabolic state of the cells as well as their response to the surrounding environment. In this study, a group of osteogenesis-related miRNAs was selected, and most of the investigated miRNAs were upregulated when the cells were cultured as pellets in glass tubes. This result could also be related to potential H3K4me3 enrichment [64]. While miR-15, miR-20a, and miR-148b enhance osteogenesis, other miRNAs, including miR-31, miR-125b and miR-133, miR-138, and miR-214 can negatively affect bone formation [33]. For example, miR-214 targets Activating Transcription Factor 4 (ATF4), which interacts with Runx2 through particular AT-rich sequence-binding protein 2 (SATB2) to regulate the expression of target genes [65, 66]. To confirm the role reported in the literature, MG63 cells were transfected with anti-miR-214, and the cells were cultured in control or osteogenic media. ALP activity was enhanced with both types of media, in comparison to the negative control or cells transfected with scrambled miRNA sequence, as shown by ALP staining. Unfortunately, the quantification of staining was below the statistical significance limit. Guo et al. (2017) reported similar findings and explained their observation by inhibiting the JNK and p38 pathways [67].

Similarly, miR-133a targets 3′UTR of *Runx2* transcript and downregulates Runx2 at both mRNA and protein levels [68]. In this study, increased miRNA-133a was associated with a decreased *Runx2* in cells cultured in glass tubes. It is noteworthy that 5-Aza-dC enhanced *Runx2* expression and inhibited that of miR-133a on glass. On the other hand, miR-125b can indirectly act on Runx2 during the early stages of osteogenesis by targeting Cbfb, a cofactor enhancing Runx2 activity [29, 69]. Furthermore, miR-138 can target FAK and block Runx2 phosphorylation by the FAK-ERK1/2 signaling pathway, which positively regulates the target genes [70]. Likewise, miR-31, a multi-target miRNA, can decrease Runx2 expression through binding to the 3′UTR region of SATB2 mRNA and inhibition of translation, which negatively influences the final effect of Runx2 [71].

Upregulation of mir-148, mir-20a, and mir-31 may activate BMP2 signaling pathway by targeting 3′UTR of the BMP antagonists Noggin, Bambi/Crim1, and smad6 mRNA, respectively, and suppress their proteins expression. Noggin blocks BMP2 binding to cognate receptors, while Bambi is a pseudo-receptor of BMP, and Crim1 tethers BMPs at the cell surface [72]. Downregulation of the two proteins allows the binding more BMP2 molecules to their functional receptors [73]. SMAD6 inhibits BMP2 receptor-mediated activation of SMAD1/5/8 (R-SMAD), which react with co-SMAD4 and translocate into the nucleus to regulate target gene transcription [74]. Finally, the results of the current study supported the potential role of the BMP2 - OSX axis for osteogenic differentiation on glass and TGF-β1- Runx2 dependent osteogenesis for those pretreated with the DNA methylation inhibitor. The limitations of this study include the endpoint analysis of differentiation rather than temporal expression. Further studies are required to investigate the hypothetical connection between different markers through their upregulation/silencing in a similar culture condition mentioned in this study. The current data suggested two distinct pathways for osteogenic differentiation, as shown by genes expression and miRNAs profiling.

## 5 Conclusion

This study investigated the combined effect of two methods for enhancing osteogenic differentiation of stem cells in a 3D pellet culture system: pre-treatment of the cells with 5-Aza-dC and culturing the cells on a glass surface during differentiation. Although both systems were associated with decreasing global DNA methylation, 5-Aza-dC pre-treatment was associated with Runx2-dependent enhancement of differentiation and general inhibition of investigated miRNA. Glass induces *OSX* and *VEGF* as well as ALP activity. Cells pretreated with 5-Aza-dC and cultured on glass showed no additive effect regarding DNA demethylation, ALP activity, or most of the studied molecular markers. This study confirmed the two models for improving the osteogenic differentiation of stem cells and would not suggest combining both systems.

## Declarations

### Author contribution statement

Latifa Alghfeli, Divyasree Parambath: Conceived and designed the experiments; Performed the experiments; Wrote the paper.

Loaa A. Tag Eldeen, Ibrahim El-Serafi: Conceived and designed the experiments; Analyzed and interpreted the data; Wrote the paper.

Ahmed T. El-Serafi: Conceived and designed the experiments; Performed the experiments; Analyzed and interpreted the data; Wrote the paper.

### Funding statement

Dr. Ahmed El-Serafi was supported by University of Sharjah (180-1090-127P).

### Data availability statement

Data included in article/supp. material/referenced in article.

### Declaration of interests statement

The authors declare no competing interests.

### Additional information

No additional information is available for this paper.

## References

[1] Khojasteh A., Hosseinpour S., Rad M.R., Alikhasi M. (2019). Buccal fat pad-derived stem cells in three-dimensional rehabilitation of large alveolar defects: a report of two cases. J. Oral Implantol..

[2] Birmingham E., Niebur G.L., McHugh P.E., Shaw G., Barry F.P., McNamara L.M. (2012). Osteogenic differentiation of mesenchymal stem cells is regulated by osteocyte and osteoblast cells in a simplified bone niche. Eur. Cell. Mater..

[3] El-Serafi A.T., Wilson D.I., Roach H.I., Oreffo R.O. (2011). Developmental plasticity of human foetal femur-derived cells in pellet culture: self assembly of an osteoid shell around a cartilaginous core. Eur. Cell. Mater..

[4] El-Serafi A.T., Hayat M. (2012). Stem Cells and Cancer Stem Cells. 8.

[5] Villagra A., Gutierrez J., Paredes R., Sierra J., Puchi M., Imschenetzky M. (2002). Reduced CpG methylation is associated with transcriptional activation of the bone-specific rat osteocalcin gene in osteoblasts. J. Cell. Biochem..

[6] Weber M., Hellmann I., Stadler M.B., Ramos L., Paabo S., Rebhan M. (2007). Distribution, silencing potential and evolutionary impact of promoter DNA methylation in the human genome. Nat. Genet..

[7] Ying Haoli, Ruolang Pan, Ye Chen (2021).

[8] El-Serafi A.T., Oreffo R.O., Roach H.I. (2011). Epigenetic modifiers influence lineage commitment of human bone marrow stromal cells: differential effects of 5-aza-deoxycytidine and trichostatin A. Differentiation.

[9] Sriram M., Sainitya R., Kalyanaraman V., Dhivya S., Selvamurugan N. (2015). Biomaterials mediated microRNA delivery for bone tissue engineering. Int. J. Biol. Macromol..

[10] Peng S., Gao D., Gao C., Wei P., Niu M., Shuai C. (2016). MicroRNAs regulate signaling pathways in osteogenic differentiation of mesenchymal stem cells (Review). Mol. Med. Rep..

[11] Alghfeli L., Parambath D., Manzoor S., Roach H.I., Oreffo R.O.C., El-Serafi A.T. (2021). Synthesis of scaffold-free, three dimensional, osteogenic constructs following culture of skeletal osteoprogenitor cells on glass surfaces. BoneKEy Rep..

[12] Fouse S.D., Shen Y., Pellegrini M., Cole S., Meissner A., Neste L.V., Jaenisch R., Fan G. (2008). Promoter CpG methylation contributes to ES cell gene regulation in parallel with Oct4/Nanog, PcG complex, and histone H3 K4/K27 trimethylation. Cell Stem Cell.

[13] Staehlke S., Rebl H., Nebe B. (2019). Phenotypic stability of the human MG-63 osteoblastic cell line at different passages. Cell Biol. Int..

[14] Choong P.F., Teh H.X., Teoh H.K., Ong H.K., Choo K.B., Sugii S. (2014). Heterogeneity of osteosarcoma cell lines led to variable responses in reprogramming. Int. J. Med. Sci..

[15] Elsharkawi I., Parambath D., Saber-Ayad M., Khan A.A., El-Serafi A.T. (2019). Exploring the effect of epigenetic modifiers on developing insulin-secreting cells. Hum. Cell.

[16] Khan A.A., Khattak M.N.K., Parambath D., El-Serafi A.T. (2021). Significant transcriptomic changes are associated with the inhibitory effects of 5-aza-2-deoxycytidine during adipogenic differentiation of MG-63 cells. Saudi J. Biol. Sci..

[17] Krattinger N., Applegate L.A., Biver E., Pioletti D.P., Caverzasio J. (2011). Regulation of proliferation and differentiation of human fetal bone cells. Eur. Cell. Mater..

[18] Abdallah B.M., Haack-Sorensen M., Fink T., Kassem M. (2006). Inhibition of osteoblast differentiation but not adipocyte differentiation of mesenchymal stem cells by sera obtained from aged females. Bone.

[19] Hera R., Keramidas M., Peoc'h M., Mouillon M., Romanet J.P., Feige J.J. (2005). Expression of VEGF and angiopoietins in subfoveal membranes from patients with age-related macular degeneration. Am. J. Ophthalmol..

[20] Xie S., Macedo P., Hew M., Nassenstein C., Lee K.Y., Chung K.F. (2009). Expression of transforming growth factor-beta (TGF-beta) in chronic idiopathic cough. Respir. Res..

[21] Hashimoto K., Kokubun S., Itoi E., Roach H.I. (2007). Improved quantification of DNA methylation using methylation-sensitive restriction enzymes and real-time PCR. Epigenetics.

[22] Livak K.J., Schmittgen T.D. (2001). Analysis of relative gene expression data using real-time quantitative PCR and the 2(-Delta Delta C(T)) Method. Methods.

[23] Liu J., Guan X., Tamura T., Ozato K., Ma X. (2004). Synergistic activation of interleukin-12 p35 gene transcription by interferon regulatory factor-1 and interferon consensus sequence-binding protein. J. Biol. Chem..

[24] Zamore P.D., Haley B. (2005). Ribo-gnome: the big world of small RNAs. Science.

[25] Li J., Xia W., Su X., Qin X., Chen Y., Li S. (2016). Species-specific mutual regulation of p53 and miR-138 between human, rat and mouse. Sci. Rep..

[26] Chai H., Liu M., Tian R., Li X., Tang H. (2011). miR-20a targets BNIP2 and contributes chemotherapeutic resistance in colorectal adenocarcinoma SW480 and SW620 cell lines. Acta Biochim. Biophys. Sin..

[27] Xu X.M., Qian J.C., Deng Z.L., Cai Z., Tang T., Wang P. (2012). Expression of miR-21, miR-31, miR-96 and miR-135b is correlated with the clinical parameters of colorectal cancer. Oncol. Lett..

[28] Vimalraj S., Partridge N.C., Selvamurugan N. (2014). A positive role of microRNA-15b on regulation of osteoblast differentiation. J. Cell. Physiol..

[29] Lin K.Y., Zhang X.J., Feng D.D., Zhang H., Zeng C.W., Han B.W. (2011). miR-125b, a target of CDX2, regulates cell differentiation through repression of the core binding factor in hematopoietic malignancies. J. Biol. Chem..

[30] Yang L., Ge D., Cao X., Ge Y., Chen H., Wang W. (2016). MiR-214 attenuates osteogenic differentiation of mesenchymal stem cells via targeting FGFR1. Cell. Physiol. Biochem..

[31] Singh S.K., Kagalwala M.N., Parker-Thornburg J., Adams H., Majumder S. (2008). REST maintains self-renewal and pluripotency of embryonic stem cells. Nature.

[32] Wu L., Yuan W., Chen J., Zhou Z., Shu Y., Ji J. (2019). Increased miR-214 expression suppresses cell migration and proliferation in Hirschsprung disease by interacting with PLAGL2. Pediatr. Res..

[33] Jing D., Hao J., Shen Y., Tang G., Li M.L., Huang S.H. (2015). The role of microRNAs in bone remodeling. Int. J. Oral Sci..

[34] Aubin J.E. (2001). Regulation of osteoblast formation and function. Rev. Endocr. Metab. Disord..

[35] Quarles L.D., Yohay D.A., Lever L.W., Caton R., Wenstrup R.J. (1992). Distinct proliferative and differentiated stages of murine MC3T3-E1 cells in culture: an in vitro model of osteoblast development. J. Bone Miner. Res..

[36] Cohen M.M. (2009). Perspectives on RUNX genes: an update. Am. J. Med. Genet..

[37] Thibault R.A., Scott Baggett L., Mikos A.G., Kasper F.K. (2010). Osteogenic differentiation of mesenchymal stem cells on pregenerated extracellular matrix scaffolds in the absence of osteogenic cell culture supplements. Tissue Eng..

[38] Hoemann C.D., El-Gabalawy H., McKee M.D. (2009). In vitro osteogenesis assays: influence of the primary cell source on alkaline phosphatase activity and mineralization. Pathol. Biol..

[39] Huang Z., Nelson E.R., Smith R.L., Goodman S.B. (2007). The sequential expression profiles of growth factors from osteoprogenitors [correction of osteroprogenitors] to osteoblasts in vitro. Tissue Eng..

[40] Lian J.B., Stein G.S., Javed A., van Wijnen A.J., Stein J.L., Montecino M. (2006). Networks and hubs for the transcriptional control of osteoblastogenesis. Rev. Endocr. Metab. Disord..

[41] El-Serafi A.T., Oreffo R.O.C., Roach H.I. (2011). Epigenetic modifiers influence lineage commitment of human bone marrow stromal cells: differential effects of 5-aza-deoxycytidine and trichostatin A. Differentiation.

[42] El-Serafi A.E.-S., Ibrahim, Castelo-Branco P.J., Carmen (2020). Histone Modifications in Therapy.

[43] El-Serafi A.T., Sandeep D., Abdallah S., Lozansson Y., Hamad M., Khan A.A. (2019). Paradoxical effects of the epigenetic modifiers 5-aza-deoxycytidine and suberoylanilide hydroxamic acid on adipogenesis. Differentiation.

[44] Tsang E.J., Wu B., Zuk P. (2018). MAPK signaling has stage-dependent osteogenic effects on human adipose-derived stem cells in vitro. Connect. Tissue Res..

[45] Ramazzotti G., Ratti S., Fiume R., Follo M.Y., Billi A.M., Rusciano I. (2019). Phosphoinositide 3 kinase signaling in human stem cells from reprogramming to differentiation: a tale in cytoplasmic and nuclear compartments. Int. J. Mol. Sci..

[46] El Shazley N., Hamdy A., El-Eneen H.A., El Backly R.M., Saad M.M., Essam W. (2016). Bioglass in alveolar bone regeneration in orthodontic patients: randomized controlled clinical trial. JDR Clin. Trans. Res.

[47] Ioannou A.L., Kotsakis G.A., Kumar T., Hinrichs J.E., Romanos G. (2015). Evaluation of the bone regeneration potential of bioactive glass in implant site development surgeries: a systematic review of the literature. Clin. Oral Invest..

[48] Zhu H., Monavari M., Zheng K., Distler T., Ouyang L., Heid S. (2022). 3D bioprinting of multifunctional dynamic nanocomposite bioinks incorporating cu-doped mesoporous bioactive glass nanoparticles for bone tissue engineering. Small.

[49] Saberi A., Behnamghader A., Aghabarari B., Yousefi A., Majda D., Huerta M.V.M. (2022). 3D direct printing of composite bone scaffolds containing polylactic acid and spray dried mesoporous bioactive glass-ceramic microparticles. Int. J. Biol. Macromol..

[50] Assis R.I.F., Schmidt A.G., Racca F., da Silva R.A., Zambuzzi W.F., Silverio K.G. (2021). DNMT1 inhibitor restores RUNX2 expression and mineralization in periodontal ligament cells. DNA Cell Biol..

[51] Kasagi S., Chen W. (2013). TGF-beta1 on osteoimmunology and the bone component cells. Cell Biosci..

[52] Matsubara T., Kida K., Yamaguchi A., Hata K., Ichida F., Meguro H. (2008). BMP2 regulates Osterix through Msx2 and Runx2 during osteoblast differentiation. J. Biol. Chem..

[53] Sepulveda H., Villagra A., Montecino M. (2017). Tet-mediated DNA demethylation is required for SWI/SNF-dependent chromatin remodeling and histone-modifying activities that trigger expression of the Sp7 osteoblast master gene during mesenchymal lineage commitment. Mol. Cell Biol..

[54] Yin C., Jia X., Miron R.J., Long Q., Xu H., Wei Y. (2018). Setd7 and its contribution to Boron-induced bone regeneration in Boron-mesoporous bioactive glass scaffolds. Acta Biomater..

[55] Zhang C., Tang W., Li Y., Yang F., Dowd D.R., MacDonald P.N. (2011). Osteoblast-specific transcription factor Osterix increases vitamin D receptor gene expression in osteoblasts. PLoS One.

[56] He S., Yang S., Zhang Y., Li X., Gao D., Zhong Y. (2019). LncRNA ODIR1 inhibits osteogenic differentiation of hUC-MSCs through the FBXO25/H2BK120ub/H3K4me3/OSX axis. Cell Death Dis..

[57] Lambrot R., Kimmins S. (2011). Histone methylation is a critical regulator of the abnormal expression of POU5F1 and RASSF1A in testis cancer cell lines. Int. J. Androl..

[58] Kondo Y., Shen L., Issa J.P. (2003). Critical role of histone methylation in tumor suppressor gene silencing in colorectal cancer. Mol. Cell Biol..

[59] Chisholm N.C., Henderson M.L., Selvamani A., Park M.J., Dindot S., Miranda R.C. (2015). Histone methylation patterns in astrocytes are influenced by age following ischemia. Epigenetics.

[60] Wang Y., Wan C., Deng L., Liu X., Cao X., Gilbert S.R. (2007). The hypoxia-inducible factor alpha pathway couples angiogenesis to osteogenesis during skeletal development. J. Clin. Invest..

[61] Diomede F., Marconi G.D., Fonticoli L., Pizzicanella J., Merciaro I., Bramanti P. (2020). Functional relationship between osteogenesis and angiogenesis in tissue regeneration. Int. J. Mol. Sci..

[62] Liu S.G., Luo G.P., Qu Y.B., Chen Y.F. (2020). Indirubin inhibits Wnt/beta-catenin signal pathway via promoter demethylation of WIF-1. BMC Complement Med Ther.

[63] Felber K., Elks P.M., Lecca M., Roehl H.H. (2015). Expression of osterix is regulated by FGF and wnt/beta-catenin signalling during osteoblast differentiation. PLoS One.

[64] Luo J., Mitra A., Tian F., Chang S., Zhang H., Cui K. (2012). Histone methylation analysis and pathway predictions in chickens after MDV infection. PLoS One.

[65] Huang X.Z., Huang J., Li W.Z., Wang J.J., Song D.Y., Ni J.D. (2020). LncRNA-MALAT1 promotes osteogenic differentiation through regulating ATF4 by sponging miR-214: implication of steroid-induced avascular necrosis of the femoral head. Steroids.

[66] Liu T.M., Lee E.H. (2013). Transcriptional regulatory cascades in Runx2-dependent bone development. Tissue Eng. B Rev..

[67] Guo Y., Li L., Gao J., Chen X., Sang Q. (2017). miR-214 suppresses the osteogenic differentiation of bone marrow-derived mesenchymal stem cells and these effects are mediated through the inhibition of the JNK and p38 pathways. Int. J. Mol. Med..

[68] Fakhry M., Hamade E., Badran B., Buchet R., Magne D. (2013). Molecular mechanisms of mesenchymal stem cell differentiation towards osteoblasts. World J. Stem Cell..

[69] Yoshida C.A., Furuichi T., Fujita T., Fukuyama R., Kanatani N., Kobayashi S. (2002). Core-binding factor beta interacts with Runx2 and is required for skeletal development. Nat. Genet..

[70] Yan X., Ehnert S., Culmes M., Bachmann A., Seeliger C., Schyschka L. (2014). 5-azacytidine improves the osteogenic differentiation potential of aged human adipose-derived mesenchymal stem cells by DNA demethylation. PLoS One.

[71] Xie Q., Wang Z., Bi X., Zhou H., Wang Y., Gu P. (2014). Effects of miR-31 on the osteogenesis of human mesenchymal stem cells. Biochem. Biophys. Res. Commun..

[72] Qureshi A.T., Doyle A., Chen C., Coulon D., Dasa V., Del Piero F. (2015). Photoactivated miR-148b-nanoparticle conjugates improve closure of critical size mouse calvarial defects. Acta Biomater..

[73] Zhang J.F., Fu W.M., He M.L., Xie W.D., Lv Q., Wan G. (2011). MiRNA-20a promotes osteogenic differentiation of human mesenchymal stem cells by co-regulating BMP signaling. RNA Biol..

[74] Wang C.J., Li B.B., Tan Y.J., Zhang G.M., Cheng G.L., Ren Y.S. (2020). MicroRNA-31/184 is involved in transforming growth factor-beta-induced apoptosis in A549 human alveolar adenocarcinoma cells. Life Sci..

